# Clastogenicity and Aneugenicity of 1,4-Benzoquinone in Different Lineages of Mouse Hematopoietic Stem/Progenitor Cells

**DOI:** 10.3390/toxics9050107

**Published:** 2021-05-12

**Authors:** Paik Wah Chow, Zariyantey Abd Hamid, Ramya Dewi Mathialagan, Nor Fadilah Rajab, Salwati Shuib, Sarina Sulong

**Affiliations:** 1 Biomedical Science Programme and Center for Diagnostic, Therapeutic and Investigative Studies, Faculty of Health Sciences, Universiti Kebangsaan Malaysia, Kuala Lumpur 50300, Malaysia; paikwah87@hotmail.com (P.W.C.); soniamirza05@hotmail.com (R.D.M.); 2 Biomedical Science Programme and Center for Healthy Ageing & Wellness, Faculty of Health Sciences, Universiti Kebangsaan Malaysia, Kuala Lumpur 50300, Malaysia; nfadilah@ukm.edu.my; 3 Department of Pathology, Faculty of Medicine, Universiti Kebangsaan Malaysia, Kuala Lumpur 56000, Malaysia; salwati@ppukm.ukm.edu.my; 4 Human Genome Center, School of Medical Sciences, Health Campus, Universiti Sains Malaysia, Kelantan 16150, Malaysia; ssarina@usm.my

**Keywords:** benzene, 1,4-benzoquinone, hematopoietic stem/progenitor cells, lineages, chromosomal aberration, Robertsonian translocation

## Abstract

Previous reports on hematotoxicity and leukemogenicity related to benzene exposure highlighted its adverse effects on hematopoiesis. Despite the reported findings, studies concerning the mechanism of benzene affecting chromosomal integrity in lineage-committed hematopoietic stem/progenitor cells (HSPCs) remain unclear. Here, we studied the clastogenicity and aneugenicity of benzene in lineage-committed HSPCs via karyotyping. Isolated mouse bone marrow cells (MBMCs) were exposed to the benzene metabolite 1,4-benzoquinone (1,4-BQ) at 1.25, 2.5, 5, 7, and 12 μM for 24 h, followed by karyotyping. Then, the chromosomal aberration (CA) in 1,4-BQ-exposed hematopoietic progenitor cells (HPCs) comprising myeloid, Pre-B lymphoid, and erythroid lineages were evaluated following colony-forming cell (CFC) assay. Percentage of CA, predominantly via Robertsonian translocation (Rb), was increased significantly (*p* < 0.05) in MBMCs and all progenitors at all concentrations. As a comparison, Pre-B lymphoid progenitor demonstrated a significantly higher percentage of CA (*p* < 0.05) than erythroid progenitor at 1.25, 2.5, and 7 μM as well as a significantly higher percentage (*p* < 0.05) than myeloid progenitor at 7 μM of 1,4-BQ. In conclusion, 1,4-BQ induced CA, particularly via Rb in both MBMCs and HPCs, notably via a lineage-dependent response. The role of lineage specificity in governing the clastogenicity and aneugenicity of 1,4-BQ deserves further investigation.

## 1. Introduction

Benzene is a common environmental pollutant and almost everyone is exposed to it via automobile exhaust, smoking, gasoline, and daily used solvent [1,2,3]. Benzene has been categorized as a Group 1 carcinogen by the International Agency for Research on Cancer in humans and animals. The studies over the years have proven that occupational and environmental exposure to benzene is associated with diseases such as aplastic anemia, myelodysplastic syndrome, and acute myeloid leukemia (AML) [1]. Benzene is a genotoxic agent and exposure to benzene is known to cause adverse effects on the bone marrow (BM) niche and altered hematopoiesis [2,4]. Numerous previous epidemiology studies demonstrated that exposure to benzene-induced genotoxic effects such as aneuploidy chromosome aberration (CA) [3,5], translocation CA [6], sister chromatid exchange [7], DNA damage [8], and alteration of gene expression level [9,10] in hematopoietic cells.

Hematopoietic niche consists of stem cells and several different lineages of progenitors which function in the hematopoiesis and production of mature blood cells such as erythrocytes, lymphocytes, monocytes, and granulocytes [11,12]. These hematopoietic stem/progenitor cells (HSPCs) work together with the regulation of intrinsic (signaling pathway or transcription factors) and extrinsic (cytokines or growth factors) factors to maintain the normal process of hematopoiesis [13]. Dysregulation of these intrinsic and extrinsic factors in HSPCs may alter hematopoiesis, resulting in cellular transformation and subsequent malignancies [14].

**Figure 1:**
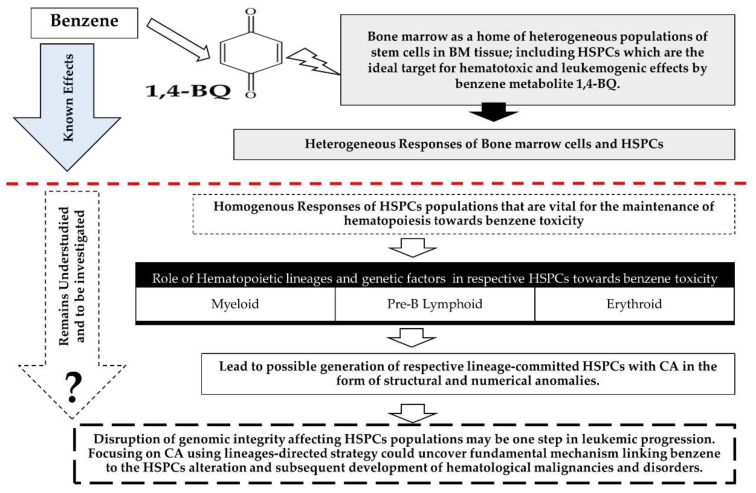
The elucidation of the genotoxicity of benzene in the respective population of lineage-committed HSPCs comprising myeloid, lymphoid and erythroid lineages focusing on CA in the form of structural and numerical anomalies. Heterogeneous responses of bone marrow cells including HSPCs towards benzene toxicity have been reported. However, homogenous responses of HSPC populations towards benzene toxicity remains unexplored and to be investigated. The study focusing on CA using lineage-directed strategy is fundamental to uncover a novel mechanism linking benzene to the disruption of genomic integrity affecting HSPCs populations and subsequent development of hematological malignancies and disorders.

A study by Wang et al. (2012) [15] speculated that benzene-mediated hematopoietic stem cells (HSCs) toxicity involved several mechanisms of action, including CA, genomic instability, macromolecular adduct formation, oxidative stress, alteration of gene expression and epigenetic regulation, apoptosis induction, error-prone DNA repair, and disruption of tumor surveillance. Moreover, leukemia is a clonal hematological malignancy initiated by epigenetic alterations or CA, either via clastogenicity (structural CA) or aneugenicity (numerical CA) occurring in HSPCs [16,17,18]. Therefore, benzene toxicity affecting the chromosomal status of HSPCs may be one of the mechanisms of benzene-induced leukemia in humans [5]. Occupational exposure to benzene was reported with the detection of aneuploidy in myeloid progenitor (CFU-GM) [3]. There was also CA such as aneuploidies and translocations detected in the lymphoid cells isolated from benzene-exposed workers’ peripheral blood samples [5]. The genomic instability caused by the genotoxic effects of benzene in progenitor cells could lead the cells to proliferate and result in the generation of pre-leukemic progenitor cells and subsequently leukemia [3]. Moreover, our previous studies showed that benzene metabolites, which are 1,4-benzoquinone (1,4-BQ) and hydroquinone (HQ), induced genotoxicity in bone marrow cells via alteration of gene-regulating self-renewing and differentiation pathways as well as chromosomal damage [19,20,21]. However, the genotoxic effect of benzene in the respective population of hematopoietic progenitors comprising myeloid, lymphoid, and erythroid remains understudied. Owing to the critical role of hematopoietic lineages and genetic factors in the maintenance of hematopoiesis, disruption of genomic integrity affecting HSPC subpopulations may be one step in leukemic progression, which justifies the importance of exploring this area of research and should be considered for further mechanistic assessment. Thus, focusing on CA using lineage-directed strategy could uncover a fundamental mechanism linking benzene to the HSPC alteration and subsequent development of hematological malignancies and disorders (Figure 1).

The study of benzene-induced toxicity in hematopoietic cells over a broad concentration range is an important approach nowadays to uncover the dose–response and mechanism of benzene-induced leukemogenesis [1,10,22]. Previous epidemiology studies demonstrated that benzene exposure caused hematotoxicity and genotoxicity on human peripheral white blood cells even at levels below the US occupational exposure limit of 1 ppm [10,22]. However, most of the previous studies in humans were focused on the higher dose or toxic concentrations of benzene exposure. Therefore, the findings regarding the effect of lower-dose benzene exposure on humans are still limited. Hiraku and Kawanishi [23] suggested that benzene metabolites at lower concentrations are more prone to induce DNA damage which leads to leukemogenesis rather than promoting cell death via apoptosis. Therefore, it is crucial to determine the genotoxic effect of benzene metabolite on the HSPCs at lower concentrations within the non-cytotoxic range in order to uncover the mechanism of benzene-induced leukemogenesis. Benzene induces its toxicity through its metabolism in the bone marrow, and one of its metabolites, 1,4-BQ, has been reported as the most reactive benzene metabolite to cause bone marrow toxicity and hematotoxicity, via oxidative stress and genotoxicity mechanisms [19,21]. Hence, the benzene metabolite 1,4-BQ was used throughout this study. The combined toxicogenomic and lineage-directed strategy was used in this study to understand the mechanism of benzene-induced leukemogenesis through CA focusing on clastogenicity (structural CA) or aneugenicity (numerical CA) evaluation in different lineages of HPCs.

## 2. Materials and Methods

### 2.1. Materials

The media and reagents used to culture BM were Dulbecco’s modified Eagle’s medium (DMEM; Invitrogen Corporation, Carlsbad, CA, USA), fetal bovine serum (FBS; JRS Scientific Inc., Woodland, CA, USA), penicillin/streptomycin (PAA Laboratories GmbH, Pasching, Australia), stem cell factor (SCF), interleukin-3 (IL-3), and interleukin-6 (IL-6) (Miltenyi Biotec, Bergisch Gladbach, Germany). The reagent, 1,4-BQ (CAS No: 106-51-4 and ≥ 98% purity) was obtained from Sigma-Aldrich Co. (St. Louis, MO, USA). Methylcellulose culture media were purchased from StemCell Technologies (Vancouver, BC, Canada) for culturing each progenitor lineages. The reagents used throughout the conventional karyotyping experiments were KaryoMAX Colcemid Solution, Trypsin EDTA 1× (Gibco, Grand Island, NY, USA), and Leishman stain (Sigma-Aldrich Co., St. Louis, MO, USA).

### 2.2. Isolation and Culturing of MBMCs

The use of mice in this study was approved by the Universiti Kebangsaan Malaysia Animal Ethics Committee with the approval number FSKB/BIOMED/2011/ZARIYANTEY/21-JULY/384-JULY-2011-MAY-2014-AR-CAT2 (date approval: 29 July 2011). Imprinting control region-strained (ICR) mice (10 weeks old, male) were purchased from the Laboratory Animal Resource Unit, Faculty of Medicine, Universiti Kebangsaan Malaysia, Kuala Lumpur, Malaysia. BM cells were isolated through femur and tibia flushing [19,24,25]. Collected BM cells were filtered through a 40 μM nylon mesh cell strainer (BD Biosciences, San Diego, CA, USA) and suspended in DMEM medium supplemented with 10% FBS, 1% penicillin/streptomycin, 100 ng/mL stem cells factor (SCF), 10 ng/mL interleukin-6 (IL-6), and 5 ng/mL interleukin-3 (IL-3) [26,27,28]. BM cells were cultured in a humidified incubator at 37 °C and 5% CO_2_ overnight prior to the experiment.

### 2.3. Treatment of MBMCs with 1,4-BQ

Stock solution of 1,4-BQ (50 mM) was freshly prepared by dissolving the 1,4-BQ powder in phosphate buffer saline (PBS). MBMCs (1 × 10^6^ cells/mL) were treated with 1.25, 2.5, 5, 7, and 12 µM of 1,4-BQ for 24 h at 37 °C and 5% CO_2_. Untreated cells were served as a negative control. These concentrations were selected based on the cytotoxicity of 1,4-BQ, as determined previously via an MTT assay [26]. The inhibitory concentration of 10% cell viability (IC_10_), inhibitory concentration of 25% cell viability (IC_25_), and inhibitory concentration of 50% cell viability (IC_50_) were 5, 7, and 12 μM, respectively. Additionally, the concentrations of 1.25 and 2.5 μM were the non-cytotoxic concentrations, as described in our previous study [19]. Etoposide with the concentration 0.05 μg/mL was used in this assay as a positive control.

### 2.4. Enrichment of Erythroid, Myeloid, and Pre-B Lymphoid Progenitor Cells Using Colony-Forming Cell (CFC) Assay

Methylcellulose semi-solid culture media with specialized formulations of growth factors (StemCells Technologies, Vancouver, BC, Canada) were used to support the growth of specific subtypes of HPCs. Three main groups of progenitors were selectively cultured including erythroid (colony-forming unit-erythroid (CFU-E) and mature burst-forming unit-erythroid (BFU-E) (MethoCult media #03334)), myeloid (colony-forming unit-granulocyte macrophage (CFU-GM), colony-forming unit-granulocyte (CFU-G), colony-forming unit-macrophage (CFU-M) (MethoCult media #03534)), and Pre-B lymphoid (colony-forming unit-Pre-B (CFU-Pre-B) (MethoCult #03630)). The CFC assay was performed according to the manufacturer’s instructions (StemCells Technologies). In brief, the 24 h treated or untreated MBMCs were collected and then centrifuged. Subsequently, cell counting was performed to prepare 100 µL of cell suspension with a viable cell number of 1 × 10^5^, 2 × 10^4^, and 5 × 10^4^ for erythroid, myeloid, and Pre-B lymphoid CFU assay, respectively. Next, the cell suspension was mixed evenly with 1 mL of progenitor-specific methylcellulose culture media and then transferred into a 6-well plate. The cells were cultured at 37 °C with 5% CO_2_ for 7 days (CFU-E, mature BFU-E and Pre-B lymphoid) and 14 days (CFU-GM, CFU-G and CFU-M) as per recommended procedure (StemCells Technologies, Vancouver, BC, Canada). Myeloid progenitors require approximately 14 days to fully develop into colonies that can be morphologically recognized and well differentiated for respective myeloid series which are granulocytes and macrophage. Meanwhile, the erythroid progenitors (CFU-E and mature BFU-E) and Pre-B lymphoid progenitor were harvested at Day 7, as beyond this time point, the quality of colonies will be compromised due to depletion of essential nutrients and pH changes resulting from the accumulation of cellular metabolic products.

### 2.5. Slide Preparation for Cytogenetic Analyses on 1,4-BQ-Treated MBMCs

Cytogenetic analysis was conducted using a method described by [20]. Colcemid (Gibco, Grand Island, NY, USA) was added to untreated and 1,4-BQ-treated cells at the final concentration 0.1 µg/mL for 3 h before being harvested. Upon harvesting, the cells were collected by centrifugation at 2500 rpm for 7 min and followed by 20 min incubation with 0.075 M KCl hypotonic solution in a 37 °C water bath. The cells were centrifuged at 2500 rpm for 5 min and fixed in 2 mL fixative (methanol: acetic acid, 3:1). The suspended cells were dropped onto pre-cleaned microscopic slides and then stained with Leishman’s stain.

### 2.6. Slide Preparation for Cytogenetic Analyses on 1,4-BQ-Treated HPCs

The progenitor-specific colonies were developed after 7 or 14 days of incubation. The cells were arrested in metaphase by colcemid treatment where 0.05 µg/mL of colcemid in the 3 mL of DMEM was added into 6-well plates with colonies and further incubated overnight before being harvested. Upon harvesting, the cells were centrifuged at 2500 rpm for 7 min and followed by 20 min of incubation with 0.075 M KCl hypotonic solution in a 37 °C water bath. The cells were centrifuged at 2500 rpm for 5 min and fixed in 2 mL fixative (methanol: acetic acid, 3:1). The suspended cells were dropped onto pre-cleaned microscopic slides and then stained with Leishman’s stain.

### 2.7. Cytogenetic Analyses on 1,4-BQ-Treated MBMCs and HPCs Using GenASIs Bandview Software

Twenty well-spread of metaphases from the prepared slides were scored for each experimental group of cells (1,4-BQ-treated and control groups). Briefly, the slides were observed under a light microscope and each metaphase was captured by a camera that was attached to the microscope. Next, the images of the chromosomes were analyzed by using GenASIs Bandview Software (Applied Spectral Imaging, Carlsbad, CA, USA). The chromosomes with the total number of 40 including the chromosome X and Y were classified as normal chromosomes and represented as 40, XY. On the other hand, the chromosome was classified as CA when one of the following CA composed of Rb (structural CA), hyperploidy (numerical CA), and complex (presence of both structural and numerical CA) was detected. The percentage of CA was calculated as per the following formula:

(1) $$\frac{\mathrm{Total}\;\mathrm{CA}}{\mathrm{Total}\;\mathrm{metaphase}\;}\;\times \;100\%$$

### 2.8. Statistical Analysis

Twenty metaphases from the prepared slides were microscopically examined and scored for each experimental group (1,4-BQ-treated and negative control groups). Each experiment was repeated thrice independently. The data are presented as means ± standard error of mean. Statistical analysis was conducted using SPSS version 16.0 (IBM Corporation, Armonk, NY, USA), and *p* < 0.05 was considered statistically significant. One-way ANOVA was employed to test the percentage of CA between the 1,4-BQ-treated and negative control groups.

## 3. Results

### 3.1. Clastogenicity and Aneugenicity of 1,4-BQ-Exposed MBMCs

**Figure 2:**
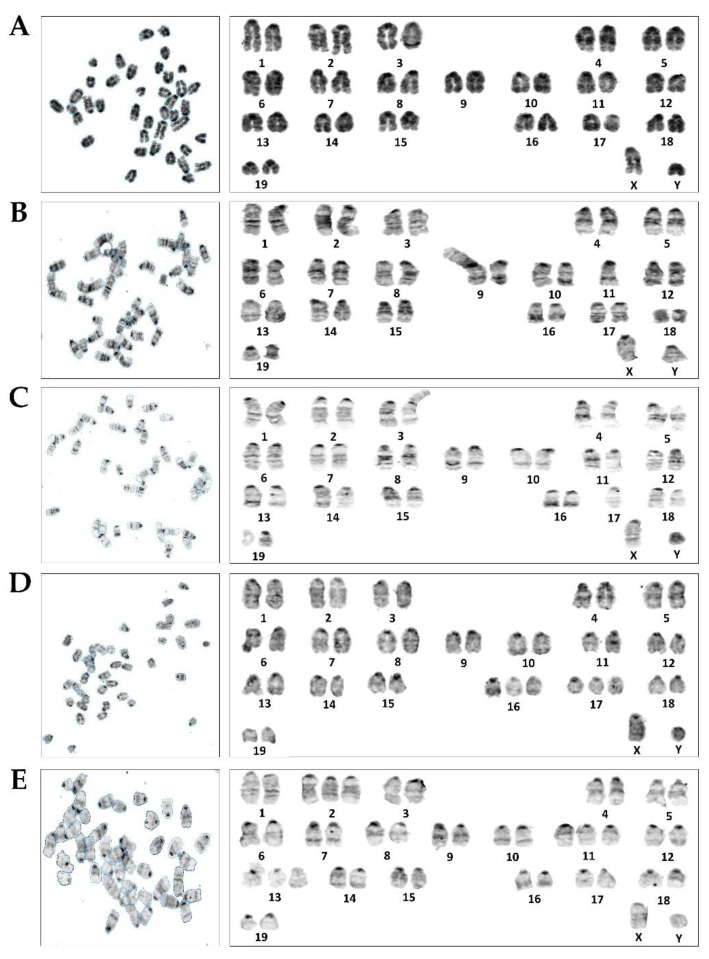
The spread and the karyotyped metaphase chromosomes of the negative control (**A**), the positive control, which is Etoposide-induced-Rb (**B**), 1,4-BQ-induced-Rb (**C**), 1,4-BQ-induced-hyperploidy (**D**), and the 1,4-BQ-induced-complex (**E**).

**Figure 3:**
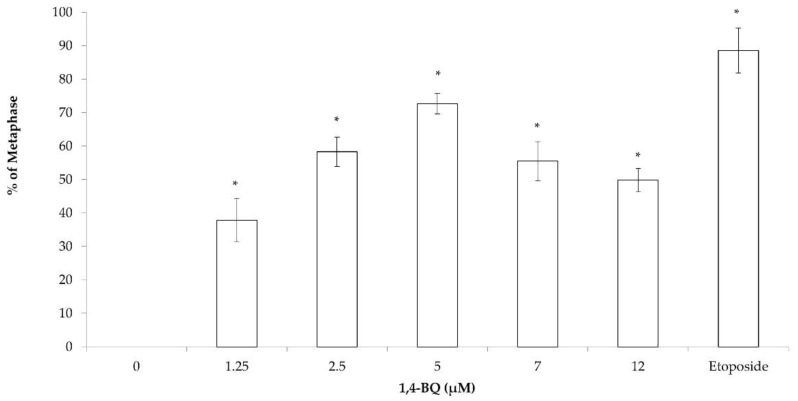
CA of MBMCs to 1,4-BQ treatment for 24 h. Twenty metaphases were scored for each experimental group and the percentage of CA is as presented. Data are presented as the mean ± SEM (*n* = 3) and analyzed by using one-way ANOVA. * *p* < 0.05 compared with the negative control group.

The cytogenetic status of 1,4-BQ-treated MBMCs was evaluated using the karyotyping assay after 24 h of exposure at 1.25, 2.5, 5, 7, and 12 μM. Figure 2 shows the spread of chromosome metaphase and arrangement of chromosomes (1-19 pairs and XY) using the karyotyping software. These concentrations were chosen based on the previous cytotoxicity assessment of 1,4-BQ on MBMCs [26]. The concentrations of 5, 7, and 12 μM of 1,4-BQ were IC_10_, IC_25_, and IC_50_, respectively, while 1.25 and 2.5 μM were the concentrations of 1,4-BQ, which were not cytotoxic on MBMCs. Etoposide with the concentration 0.05 μg/mL was used in this assay as a positive control. Exposure to 1,4-BQ caused CA in MBMCs at all tested concentrations significantly (Figure 3) compared with the negative control group. The abnormalities of chromosomes were increased from 1.25 to 5 μM, but then reduced from 7 to 12 μM. The highest percentage of CA was noted at 5 μM 1,4-BQ-induced MBMCs.

**Figure 4:**
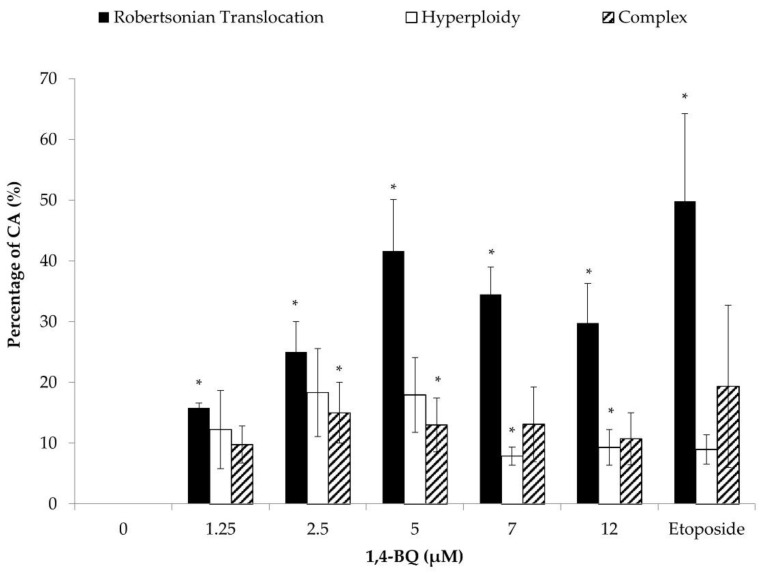
Effect of 1,4-BQ on different types of CA (Rb, hyperploidy, and complex) in MBMCs after 24 h of treatment with 1,4-BQ. Twenty metaphases were scored for each experimental group, and the percentage of each type of CA is as presented. Data are presented as the mean ± S.E.M. (*n* = 3) and analyzed by using one-way ANOVA. * *p* < 0.05 compared with the negative control group.

Each of the metaphases was karyotyped and the CA was classified according to the types of abnormalities, which were Rb, hyperploidy, and complex. Exposure to 1,4-BQ predominantly induced Rb in MBMCs as this biomarker was more significantly increased at all tested concentrations of 1,4-BQ relative to hyperploidy and complex parameters (Figure 4). However, 1,4-BQ only caused a significant higher percentage of hyperploidy and complex in MBMCs at particular concentrations.

### 3.2. Clastogenicity and Aneugenicity of 1,4-BQ-Exposed Myeloid Progenitor Cells

**Figure 5:**
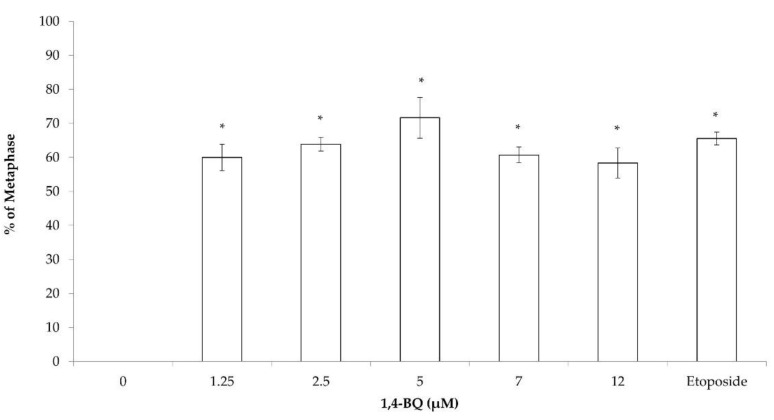
CA of myeloid progenitor cells harvested after 14 days of CFC assay following pre-exposure of MBMCs to 1,4-BQ for 24 h. Twenty metaphases were scored for each experimental group and the percentage of CA is as presented. Data are presented as the mean ± S.E.M. (*n* = 3) and analyzed by using one-way ANOVA. * *p* < 0.05 compared with the negative control group.

**Figure 6:**
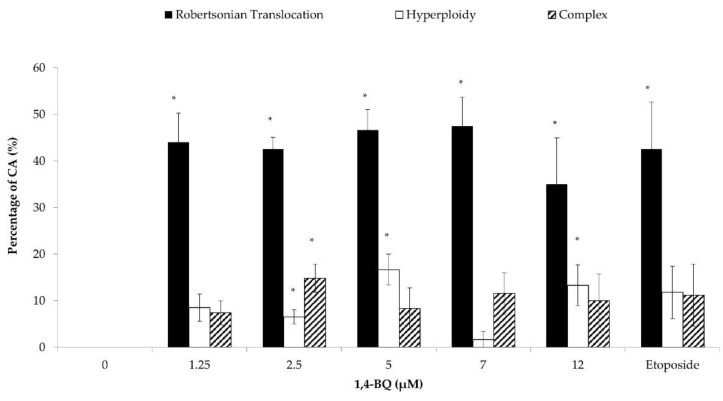
Effect of 1,4-BQ on different types of CA (Rb, hyperploidy, and complex) in myeloid progenitor cells harvested after 14 days of CFU assay following pre-exposure of MBMCs to 1,4-BQ for 24 h. Twenty metaphases were scored for each experimental group and the percentage of each type of CA is as presented. Data are presented as the mean ± S.E.M. (*n* = 3) and analyzed by using one-way ANOVA. * *p* < 0.05 compared with the negative control group.

The effect of 1,4-BQ on the cytogenetic status of each type of HPCs was evaluated using the karyotyping assay after 24 h of exposure at 1.25, 2.5, 5, 7, and 12 μM. The 1,4-BQ-treated MBMCs were cultured in methylcellulose agar (MethoCult media #03534) for 14 days to form the CFU-GM, CFU-G, and CFU-M and then were harvested for karyotyping assay. Exposure to 1,4-BQ caused CA on myeloid progenitor cells at all tested concentrations significantly (Figure 5). The percentage of abnormal metaphase was increased from 1.25 to 5 μM, but reduced from 5 to 12 μM. The highest percentage of CA was noted at 5 μM 1,4-BQ-induced myeloid progenitors. Each of the CA was further karyotyped. Exposure to 1,4-BQ predominantly induced Rb in myeloid progenitor cells as it was significantly increased in Rb at all tested concentrations of 1,4-BQ (Figure 6).

### 3.3. Clastogenicity and Aneugenicity of 1,4-BQ-Exposed Pre-B Lymphoid Progenitor Cells

**Figure 7:**
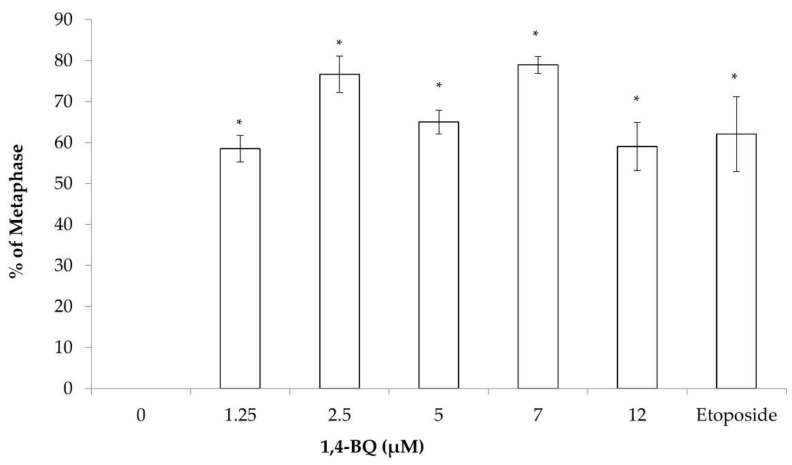
CA of Pre-B lymphoid progenitor cells harvested after 7 days of CFU assay following pre-exposure of MBMCs to 1,4-BQ for 24 h. Twenty metaphases were scored for each experimental group and the percentage of CA is as presented. Data are presented as the mean ± S.E.M. (*n* = 3) and analyzed by using one-way ANOVA. * *p* < 0.05 compared with the negative control group.

**Figure 8:**
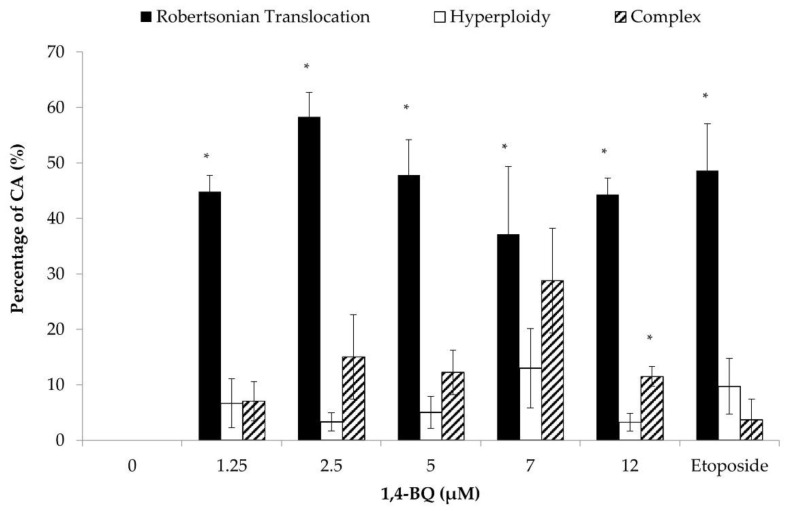
Effect of 1,4-BQ on different types of CA (Rb, hyperploidy, and complex) in Pre-B lymphoid progenitor cells harvested after 7 days of CFU assay following pre-exposure of MBMCs to 1,4-BQ for 24 h. Twenty metaphases were scored for each experimental group and the percentage of each type of CA is as presented. Data are presented as the mean ± S.E.M. (*n* = 3) and analyzed by using one-way ANOVA. * *p* < 0.05 compared with the negative control group.

The colony-forming unit-Pre-B lymphoid cells from methylcellulose agar (MethoCult media #03630) were harvested for karyotyping assay after 7 days of culture. Exposure to 1,4-BQ caused CA on Pre-B lymphoid progenitor at all tested concentrations significantly (Figure 7) and induced Rb at all tested concentrations of 1,4-BQ significantly (Figure 8).

### 3.4. Clastogenicity and Aneugenicity of 1,4-BQ-Exposed Erythroid Progenitor Cells

**Figure 9:**
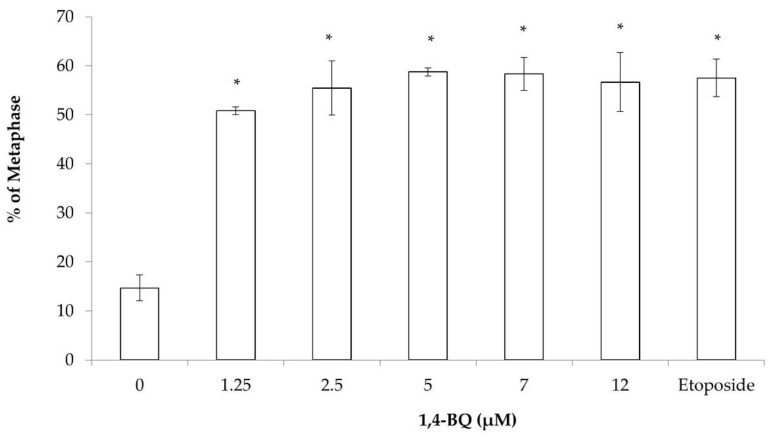
CA of erythroid progenitor cells harvested after 7 days of CFU assay following pre-exposure of MBMCs to 1,4-BQ for 24 h. Twenty metaphases were scored for each experimental group and the percentage of CA is as presented. Data are presented as the mean ± S.E.M. (*n* = 3) and analyzed by using one-way ANOVA. * *p* < 0.05 compared with the negative control group.

**Figure 10:**
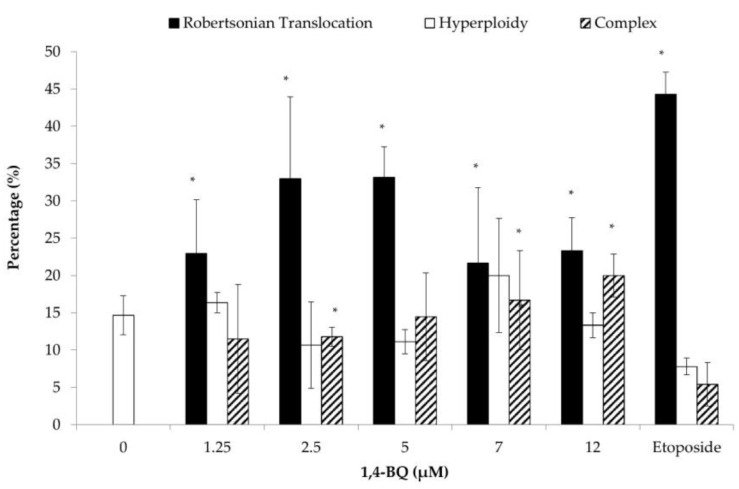
Effect of 1,4-BQ on different types of CA (Rb, hyperploidy, and complex) in erythroid progenitor cells harvested after 7 days of CFU assay following pre-exposure of MBMCs to 1,4-BQ for 24 h. Twenty metaphases were scored for each experimental group and the percentage of each type of CA is as presented. Data are presented as the mean ± S.E.M. (*n* = 3) and analyzed by using one-way ANOVA. * *p* < 0.05 compared with the negative control group.

The 1,4-BQ-treated MBMCs were cultured in methylcellulose agar (MethoCult media #03334) for 7 days to form the colony-forming unit-erythroid (CFU-E) and mature burst-forming unit-erythroid (BFU-E). The erythroid progenitor colony cells were then harvested for karyotyping assay. Exposure to 1,4-BQ caused CA on erythroid progenitor at all tested concentrations significantly (Figure 9). Each of the CA was further karyotyped. Exposure to 1,4-BQ predominantly induced Rb in erythroid progenitor, as it significantly caused Rb at all tested concentrations of 1,4-BQ (Figure 10), while it only caused complex abnormalities at 2.5, 7, and 12 μM. Hyperploidy was detected in the negative control erythroid progenitor cells at 14.7 %.

### 3.5. Differences of Cytogenetic Status between the Three Different Lineages of Hematopoietic Progenitors after 1,4-BQ Exposure

**Figure 11:**
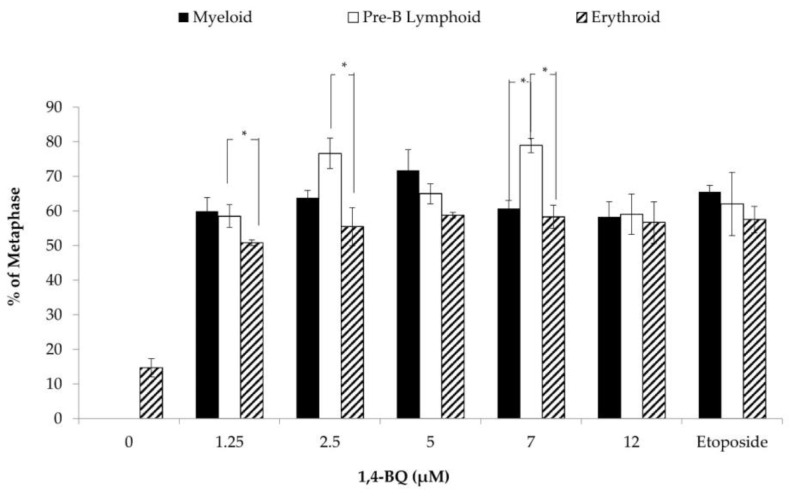
CA in different lineages of hematopoietic progenitors harvested after CFU assay following pre-exposure of MBMCs to 1,4-BQ treatment for 24 h. Twenty metaphases were scored for each experimental group and the percentage of CA is as presented. Data are presented as the mean ± S.E.M. (*n* = 3) and analyzed by using one-way ANOVA. * *p* < 0.05 compared between the progenitors.

The percentage of CA of three different lineages of HPCs (myeloid, Pre-B lymphoid, and erythroid progenitors) were compared by using the Post Hoc Tukey one-way ANOVA statistical test (Figure 11). Pre-B lymphoid progenitor has a significantly higher percentage of CA than erythroid progenitor at several concentrations of 1,4-BQ (1.25, 2.5, and 7 μM). Besides that, the percentage of CA of Pre-B lymphoid progenitor was also significantly higher than myeloid progenitor at 7 μM 1,4-BQ.

## 4. Discussion

The mechanism of benzene-induced hematological diseases and malignancies is still not fully understood. Many previous studies demonstrated that the main target of benzene-induced toxicity is HSPCs [19,29,30,31,32]. Few previous epidemiology studies showed the association between human exposure on benzene and CA such as translocation and aneuploidy [3,5,33]. The induction of CA is a potential mechanism underlying benzene-induced leukemogenesis and hematological diseases. A study by Smith (2010) [34] hypothesized that reactive benzene metabolites such as 1,4-BQ or HQ may cause CA, mitotic recombination aberration, and epigenetic alteration in HSPCs. Such events may further cause gene mutations or changes in gene expression, some of which might affect the self-renewal and differentiation of these cells. The mutated cells that survived from apoptosis will carry the altered gene variants which then may form the leukemic stem and progenitor cell clone and subsequently cause leukemia. Thus, the evaluation of the chromosomal status of HSPCs after exposure to the benzene metabolite 1,4-BQ is the main objective of this ex vivo study.

This study reveals that exposure to 1,4-BQ induced CA in MBMCs at all tested concentrations which are cytotoxic or non-cytotoxic. Our previous study [26] demonstrated that 1,4-BQ caused a cytotoxic effect in MBMCs at concentrations 5 μM and above. Previous studies reported that benzene exposure induced CA in hematopoietic cells at high and even at low levels of exposure [10,22]. Thus, both ranges of concentrations (cytotoxic and non-cytotoxic) were used in this study to determine the genotoxicity of 1,4-BQ on the chromosomal status of HSPCs. It is not surprising that 1,4-BQ caused CA in MBMCs as shown in this study, as many previous studies have reported that this benzene metabolite is a genotoxic agent [19,24,35,36,37,38]. However, our study is the first to show the induction of CA by 1,4-BQ in MBMCs even at low and non-cytotoxic concentrations.

The induction of CA in MBMCs was perhaps caused by the characteristic of 1,4-BQ as an inhibitor of topoisomerase IIα enzyme. Few previous studies have reported that 1,4-BQ caused leukemia via the mechanism of inhibiting the topoisomerase Iiα enzyme [39,40,41,42,43,44]. The inhibition of this enzyme will break the DNA double strand and thus cause DNA damage. Besides that, the topoisomerase IIα enzyme is also playing a role in chromosomal condensation and separation during the mitosis process [45]. Therefore, the inhibition of this enzyme can induce chromosomal breaks and translocations and also aneuploidy due to chromosome malsegregation that generate chromosomal instability and subsequently cause leukemia [42,46]. In this study, etoposide with the concentration 0.05 μM was used as a positive control for the CA induction in MBMCs and HPCs. Etoposide was effectively used as a chemotherapy agent that has the same action mechanism as topoisomerase IIα inhibitor [47]. A study by Choudhury et al. [48] reported that etoposide induced CA in MBMCs. Thus, in the same way as etoposide, 1,4-BQ may induce CA in treated cells through inhibiting the topoisomerase IIα enzyme and cause DNA strand breaks, which may lead to the formation of structural chromosome aberrations and possibly chromosome segregation errors during mitosis.

This study is the first study to demonstrate that 1,4-BQ induced CA in MBMCs even at non-cytotoxic concentrations (1.25 and 2.5 μM). The percentage of abnormal metaphase on MBMCs was increased from 1.25 to 5 μM, but reduced from 7 to 12 μM. These concentrations (5, 7, and 12 μM) of 1,4-BQ were shown to cause cytotoxicity and apoptosis in MBMCs [26]. This benzene metabolite is very reactive and damages the cell membrane at these high concentrations and thus induces apoptosis [49,50]. Hence, 1,4-BQ caused cell death rather than genetic defects at higher concentrations. These findings are in agreement with the previous study by Yager et al. [38], where they reported that the micronuclei frequency in human lymphocytes was not significantly increased at higher concentrations of 1,4-BQ exposure. Besides that, the study by Tung et al. [37] also demonstrated that the DNA repair ability of fetal liver cells was reduced after 24 h of exposure to 25 μM of 1,4-BQ, which caused significant cell death. Therefore, the extracellular exposure of a high concentration of 1,4-BQ may have caused cytotoxicity before entering the nucleus and exerting genotoxic effects [38,51].

Our previous study [26] reported that hematopoietic lineage plays a significant role in the 1,4-BQ-induced cytotoxicity. Thus, in this current study, we were interested to see the difference between the lineage progenitors towards the genotoxicity of 1,4-BQ. The different lineage progenitors such as myeloid, Pre-B lymphoid, and erythroid were cultured in specific methylcellulose media after exposure to 1,4-BQ for 24 h. The surviving progenitors which escaped from apoptosis or the cytotoxic effect of 1,4-BQ proliferated in the media and their chromosomal status was determined. In brief, 1,4-BQ exposure caused clastogenicity and aneugenicity in all types of progenitors at all tested concentrations significantly. Until now, there has been no study to demonstrate the 1,4-BQ toxicity on the cytogenetic status of different lineage progenitors. Many previous studies have proven that benzene toxicity is related to hematological disorders that involve different types of lineages of hematopoietic cells such as aplastic anemia (erythroid lineage), myelodysplasia syndrome (myeloid lineage), lymphoma (lymphoid lineage), and leukemia (myeloid and lymphoid lineage). Up to our knowledge, the approach to use lineage-directed strategy combined with toxicogenomic study has been used in one previous epidemiology study where they reported that benzene exposure in industry workers caused CA in the myeloid progenitors (CFU-GM) [3]. However, there is no study to show the cytogenetic status of other lineage progenitors. Therefore, the clastogenicity of 1,4-BQ on different types of lineage progenitors is an important point to be ruled out in order to understand the mechanism of the genotoxicity of benzene.

In this study, there was hyperploidy detected in the negative control erythroid progenitor cells (14.7 ± 2.6%). This finding is in agreement with the previous study by Gowans et al. [52], which also reported that negative control colony cells (CFU-spleen) from CBA-Ca and C57BL/6 mice may have CA. They cultured the MBMCs in the spleen to form the CFU-S, where they verified that the in vivo culture condition caused the cells to have proliferative stress. Thus, there is a spontaneous level of DNA damage in this progeny of HSPCs when cultured under conditions of proliferative stress [52].

Our study reveals that Rb was the predominant CA induced by 1,4-BQ compared to other types of CA (hyperploidy and complex). The natural structure of the mouse chromosome is acrocentric or telocentric. This causes the mouse chromosomes to predominantly recombine with other chromosomes at the centromere region, which is a type of CA called Rb [53]. Besides that, a previous study by Shaffer and Lupski [54] demonstrated that the human chromosomes 13, 14, 15, 21, and 22, which have an acrocentric structure, were also more sensitive to having Rb as in the event of CA. Thus, Rb predominantly happened compared to other types of CA (hyperploidy and complex) in our MBMCs and three types of progenitors. Since the chromosomes of mice are prone to Rb due to the predominance of acrocentric and telocentric structures, the relevance of this finding in humans’ chromosomes should be investigated in the future study.

Based on the evidence provided by certain previous studies, Rb is mainly caused by a few mechanisms, namely telomere inactivation, telomere shortening, breakpoint at centromere, and increased expression of c-myc oncoprotein [55,56]. In this study, the most probable mechanism that induced Rb in mouse chromosomes is telomere shortening, which was caused by the 1,4-BQ-induced oxidative stress. A previous study by Kawanishi and Oikawa [57] demonstrated that UV light exposure caused oxidative stress and the formation of 8-oxo-7,8-dihydro-2′-deoxyguanosine (8-oxodG) on the telomere sequence triplet GGG. This further resulted in telomere shortening in the chromosome of HL-60 leukemic cells. Other than that, the study by Yin et al. [58] also showed that 1,4-BQ derivatives such as tetrachloro-1,4-BQ and tetrabromo-1,4-BQ induced oxidative stress by formating the 8-oxodG in a cell-free system. Therefore, the findings of this study speculate that the oxidative stress induced by 1,4-BQ caused the DNA damage at the part of the telomere sequence triplet GGG and further led to telomere shortening and Rb.

Another plausible explanation for telomere loss induced by 1,4-BQ is due to the generation of DNA double-strand breaks (DSBs). DSBs cause the formation of terminal acentric chromosome fragments which are lost from the main nuclei during mitosis as micronuclei. This is evidenced by Abernethy et al. [35], whereby 1,4-BQ caused micronuclei formation in HSCs. Thus, the formation of the micronuclei will effectively result in the complete telomere removal from one or more of the chromosome arms in the main nuclei. The chromosomes that lose the telomeres will then fuse to form an Rb chromosome. However, to date, there is no study to demonstrate the effect of 1,4-BQ on the telomere of chromosome. Thus, this study deserves further investigation.

Few previous studies demonstrated that benzene or its metabolites caused aneuploidy specifically hyperploidy in the human blood cells. The in vitro study by Eastmond et al. (1994) showed that HQ caused hyperdyploidy on the isolated human peripheral lymphocytes [59]. In the study by Zhang et al. (1996), the peripheral blood cells from 43 workers that were occupationally exposed to benzene were analyzed using the cytogenetic method and there was a significantly increased hyperploid frequency of chromosome 9 [60]. Benzene metabolite, especially 1,4-BQ, is a potent inhibitor of microtubule assembly, whereby it can bind covalently to tubulin and disrupt the microtubules of the mitotic spindle. Eventually, it causes missegregation of chromosomes, which leads to aneuploidy.

A previous study by Hiraku and Kawanishi [23] suggested that benzene metabolites at lower concentrations are more prone to induce DNA damage which leads to leukemogenesis rather than promoting cell death via apoptosis. Thus, the fates of the cells exposed to benzene metabolites depend on the concentrations of benzene metabolites, which determine whether the cells will accumulate DNA damage or undergo apoptosis. Therefore, determining the clastogenic and aneugenic effect of benzene metabolites at a lower concentration or non-cytotoxic range in the hematopoietic niche targeting different lineages is important to uncover the mechanism of benzene-induced leukemogenesis. In our study, the findings demonstrate that benzene metabolites, such as 1,4-BQ, induced CA in MBMCs in each lineage of progenitors even at low and non-cytotoxic concentrations. Although previous studies have shown that 1,4-BQ is genotoxic to hematopoietic cells even at low concentrations [24,35,38], so far there is no study showing the effect of 1,4-BQ on the cytogenetic status of each lineage of HPCs. For example, the study by Tian et al. [25] reported that the gene expression of *p15* in MBMCs was significantly reduced after exposure to 1 μM of 1,4-BQ. Apart from that, a study by Abernethy et al. [35] demonstrated that exposure to 1,4-BQ at 5 μM of 1,4-BQ (which was not cytotoxic to human HSCs) induced a micronucleated cell in CD34^+^ cells significantly. Our previous study [19] also demonstrated that the low, yet non-cytotoxic, concentration of 1,4-BQ significantly increased the gene expression of self-renewal (*HoxB4*) and differentiation (*GATA3*) genes in HSPCs. On the other hand, the previous epidemiology study by McHale et al. [9] reported that even low levels of benzene exposure could induce significant perturbation of gene expression in the Acute Myeloid Leukemia (AML) and immune response pathway in the peripheral blood mononuclear cells of benzene-exposed workers. Moreover, a genotoxic effect such as genomic instability that may induce mutations that give cells a survival or proliferation advantage could lead to the generation of pre-leukemic stem or progenitor cells and induce leukemia [3]. Thus, the clastogenicity and aneugenicity of 1,4-BQ-exposed HPCs at low concentrations is the novel finding from our study that contributes to a better understanding of the mechanism of benzene-induced leukemogenesis.

As a comparison between the three different lineages of progenitors, Pre-B lymphoid progenitor showed a significantly higher percentage of CA than erythroid progenitor at three concentrations of 1,4-BQ exposure, which were 1.25, 2.5, and 7 μM. Moreover, the percentage of CA of Pre-B lymphoid progenitor was also significantly higher than myeloid progenitor at 7 μM. To our knowledge, there is no study before to compare the cytogenetic status between different lineages of progenitors upon exposure with benzene or its metabolites. However, many previous studies have proven that benzene toxicity is associated with hematological disorders that involve different types of lineages of hematopoietic cells, such as aplastic anemia (erythroid lineage), myelodysplasia syndrome (myeloid lineage), lymphoma (lymphoid lineage), and leukemia (myeloid and lymphoid lineage). Hence, it is prudent to point out that the benzene metabolite 1,4-BQ induced clastogenicity and aneugenicity in mouse HPCs are dependent on which lineages are investigated. The difference of susceptibility of these different lineages towards the genotoxicity of 1,4-BQ may be due to the differences in detoxification and antioxidant defense mechanism of cells from different hematopoietic lineages. These novel findings on benzene-induced clastogenicity and aneugenicity in HPCs need to be verified in further studies that should also explore the mechanisms that determine the sensitivity of different progenitor cell lineages towards the clastogenicity and aneugenicity of 1,4-BQ.

## 5. Conclusions

In conclusion, the benzene metabolite 1,4-BQ induced clastogenicity and aneugenicity in MBMCs and three different lineages of progenitors, which are myeloid, Pre-B lymphoid, and erythroid progenitors. This CA induction happened even at low, yet non-cytotoxic, concentrations, highlighting the risk of HPCs acquiring genetic damage at low concentrations of 1,4-BQ exposure and the subsequent development of blood pathology such as aplastic leukemia, MDS, and leukemogenesis. Moreover, 1,4-BQ induced Rb at a higher frequency compared to other types of CA which are hyperploidy and complex. Then, Pre-B lymphoid progenitor showed a higher percentage of CA than erythroid and myeloid progenitors at several concentrations of 1,4-BQ exposure. The overall findings of this study provide new insight into the benzene-induced genotoxicity of the hematopoietic niche with the different lineages’ involvement.

## Data Availability

Not applicable.
